# Determinants for acceptance of preventive treatment against heart disease – a web-based population survey

**DOI:** 10.1186/1471-2458-14-783

**Published:** 2014-08-02

**Authors:** Nielsen Jesper Bo, Jarbøl Dorte Ejg, Gyrd-Hansen Dorte, Barfoed Benedicte Marie Lind, Larsen Pia Veldt

**Affiliations:** Research Unit of General Practice, Institute of Public Health, University of Southern Denmark, J.B.Winsløws Vej 9, DK-5000 Odense C, Denmark; COHERE, Institute of Public Health, University of Southern Denmark, J.B.Winsløws Vej 9, DK-5000 Odense C, Denmark

**Keywords:** Acceptance, Preventive treatment, Heart disease, Determinants, Population survey, Risk communication, Socioeconomics, Side effects, Cost of treatment

## Abstract

**Background:**

Patients’ perception of risk and their lifestyle choices are of major importance in the treatment of common chronic diseases. This study reveals determinants for and knowledge about why people accept or reject preventive medical interventions against heart disease.

**Methods:**

A representative sample of 40-60-year-old Danish inhabitants was invited to participate in a web-based survey. The respondents were presented with a hypothetical scenario and asked to imagine that they were at an increased risk of heart disease, and subsequently presented with an offer of a preventive medical intervention. The aim was to elicit preference structures when potential patients are presented with different treatment conditions.

**Results:**

About one third of the respondents were willing to accept preventive medical treatment. Respondents with personal experience with heart disease were more likely to accept treatment than respondents with family members with heart disease or no prior experience with heart disease. The willingness to accept treatment was similar for both genders, and when adjusting for experience with heart disease, age was not associated with willingness to accept treatment. Socioeconomic status in terms of lower education was positively associated with acceptance. The price of treatment reduced willingness to accept for the lower income groups, whereas it had no effect in the highest income group. Some 57% of respondents who were willing to accept treatment changed their decision following information on potential side effects.

**Conclusions:**

In accordance with our pre-study hypothesis, individuals with low income were more sensitive to price than individuals with high income. Thus, if the price of preventive medication increases above certain limits, a substantial proportion of the population may refrain from treatment. More than half of the respondents who were initially willing to accept treatment changed their decision when informed about the presence of potential side effects. This is an important observation in relation to risk communication, since most side effects occur very seldom, and a skewed assessment of treatment efficacy compared to risk of side effects may refrain some patients from treatment. Thus, more research is needed to better allow patients to compare treatment efficacy with risk of side effects in quantitative terms.

## Background

Vast resources are spent on developing new treatment regimes, but if patients are unwilling to accept or adhere to the treatment, the investment will have been in vain. Patients’ perception of risk and their lifestyle choices are of major importance for prevention and early treatment of common chronic diseases, such as type 2 diabetes, hypertension and chronic obstructive lung disease, where clinical health effects are often few or absent in the early stages of the disease. Acceptance is seen as the initial decision by the patient to accept a treatment plan, adherence is whether the patient actually begins medication or lifestyle change, and persistence is whether the patient continues an agreed treatment plan for a longer period of time. The present study will enhance knowledge about possible determinants for acceptance and rejection of preventive interventions related to medical treatment.

Chronic diseases represent a significant burden to society in terms of number of patients and treatment costs. Lifestyle diseases are expected to account for 70% of all chronic diseases by 2020, and it has been estimated that lifestyle changes and medical interventions can prevent or delay 80% of all heart diseases, strokes and cases of diabetes, and 40% of all cancers [1]. Clinical experience, however, suggests that lack of adherence significantly reduces the efficacy of such preventive interventions [2–4]. In a long-term study on persistence of statin use, persistence was evaluated separately in patients without clinical indications for cardiovascular disease (primary prevention, n = 136 000) and in patients with coronary artery disease (secondary prevention, n = 94 000) [5]. This study demonstrated that persistence was significantly lower for the primary prevention group (45%) compared to the secondary prevention group (59%). In both groups more than 75% of the patients stopped taking statins within 2 years [5]. Comparable numbers have been observed in other studies on persistence of statin use, and predictors of poor long-term persistence included older age, cardiovascular morbidity, and low income [6].

Preventive interventions strongly depend on the ability and inclination of an individual to accept and adhere to an agreed medical treatment. Studies based on the health belief model have demonstrated that beliefs, concerns, and perceived necessity of treatment play an important role in adherence [7]. Patients’ acceptance is likely to drive their behaviour towards their treatment, i.e. their adherence and persistence. Thus, acceptance differs from adherence, as it predicts the patient’s behaviour, while adherence is the patient’s current behaviour towards their treatment [8]. This indicates that measuring patients’ acceptance is likely to predict their later adherence and persistence with treatment.

Willingness to accept preventive medications and adherence to treatment depend on how the person at risk perceives benefits, potential side effects, costs, as well as interactions between these factors [8, 9]. Reluctance to accept and adhere to a treatment may appear particularly tempting when the investment (e.g. money or use of preventive medication) is needed in the near future, whereas the health gains are incurred as a risk reduction years ahead [4, 10]. Time lost due to frequent GP visits may also be seen as a treatment-related cost for the patient.

Investment in education is likely to be associated with a low discount rate (time preference) as it is associated with present investment (costs) and gains in the distant future [11]. This resembles the choice scenario for a preventive treatment like the one in the present study – merely in the context of health investment. Under the premise that time preference rates are similar in the context of health and education one may expect that individuals with higher education are more willing to accept/invest in a preventive treatment. Although empirical work by van der Pol [12] suggests that the time preference rates across these settings may be quite different, it is the *ex ante* hypothesis of this paper that respondents with higher levels of education are more likely to invest in future health, and that this association is likely driven by a lower time preference rate amongst those who are highly educated.

Besides personal experience with a given disease, knowledge and experience of family members and friends have previously been shown to correlate with adherence [in 10]. A more recent interview-based survey on hypothetical scenarios confirmed that personal experience with cardiovascular disease affected the acceptance of medication, whereas this was not the case if family members had experienced a cardiovascular disease [13]. However, the latter study included non-symptomatic risk factors for heart disease (e.g. hypertension, hypercholesterolemia), which may have diluted the degree of concern and therefore the inclination to accept treatment.

Identifying the potential for societal welfare gains from disease prevention requires an understanding of what people at risk value when making their choices, and why they value certain factors more than others. The most important determinants of health are related to individual behaviour, including individuals’ choices and trade-offs that may significantly affect their health [14, 15].

The hypothesis in the present study is that willingness to accept a preventive treatment of heart disease is influenced by cost, experience with heart disease, age, educational attainment, frequency of needed control visits to the GP, and the presence of potential side effects from the treatment.

The specific hypotheses are:

1. Individuals with personal or family-related experience with heart disease are more willing to accept treatment.
2. Individuals with low income are more sensitive to the price of treatment than individuals with high income.
3. Individuals with higher education are more willing to accept/invest in a preventive treatment.
4. Increased frequency of control visits to the GP reduces the willingness to accept treatment.
5. The decision to accept treatment is influenced by the presence of a risk of side effects.
6. Individuals with personal experience with heart disease are more willing to accept treatment despite potential side effects than individuals with no prior experience with heart disease.

## Methods

### Sample and setting

A representative sample of Danish speaking inhabitants of Denmark aged 40–60 years were invited to participate in the survey. In the survey, the respondents were presented with a hypothetical scenario and asked to imagine that they were at an increased risk of heart disease, and subsequently presented with an offer of a preventive medical intervention targeted at reducing the risk of heart disease. Treatment benefit was given as an absolute risk reduction (from 10% to 5%). The aim was to elicit preference structures when potential patients are presented with different treatment conditions.

Data were collected through a web-based questionnaire using TNS Gallup’s web-panel GallupForum. The survey ran between 15 and 22 March 2012. Among the panel members who accessed the website, 91% answered the questionnaire. The age group from 40–60 years was considered relevant for first time users of preventive therapy against cardiovascular disease.

According to the Act on a Biomedical Research Ethics Committee System, the project was not a biomedical research project and therefore did not need the ethic committee’s approval.

### Questionnaire

The questionnaire included a description of a risk scenario together with 12 questions concerning health, lifestyle and willingness to accept treatment under various conditions, plus a number of questions on sociodemographic characteristics (gender, age, self-rated health status, highest educational attainment, and household income). The present paper focuses on the four questions related to willingness to accept a treatment and personal experience with heart disease (see section on Extract of questionnaire). The two initial questions (regarding self-rated health and experience with heart disease (personal or in the family)) have been used in previous studies [13], whereas the latter two were specifically developed for this study. Prior to being presented to the web-panel, the entire questionnaire was evaluated regarding comprehensibility, relevance, acceptability and feasibility, and pilot tested by TNS Gallup. Two variables on treatment conditions (price of medication and frequency of visits to the GP) were varied over three levels. Each respondent was presented with only one treatment option when asked whether he or she would accept the treatment (see section on Extract of questionnaire). The design was fully factorial, which means that the impact of the two types of costs can be analysed independently.

### Extract of questionnaire

1. How would you rate your present state of health in general?
  - Very good
  - Good
  - Fair
  - Bad
2. Do you have knowledge of any heart disease of your own or within your family?

- Yes, I have a heart disease myself
- Yes, I have had a heart disease myself
- Yes, there are others in my family who have or have had a heart disease
- No
  Imagine that you are visiting your GP.
  The GP tells you that you have an increased risk of a heart disease even though you presently do not experience any troublesome symptoms.
  Your GP informs you that for one in ten persons like you, the disease will develop and have serious consequences for your health.
  You cannot know beforehand whether you belong to the small group (10%) who will get the heart disease, or to the larger group (90%) who will not.
  There is now a possibility of medical treatment.
  The medicine is preventive, and you will need to take it the rest of your life.
  When you begin taking the medicine it will immediately reduce your risk of serious heart disease from 10% to 5%.
  The medicine will cost you X DKK every month (X = 100, 400, 700), and you will need to visit your GP for control Y times (Y = 2 times per month, 1 time per month, 4 times per year).

- Will you based on this information accept the offer and begin preventive medical treatment?
- Yes
- No

Related to the use of the medicine some people will for shorter or longer periods experience side effects such as lack of appetite, dizziness, tiredness, headache, and an increased tendency to sweat. Often these side effects may occur before you will get the benefit of the medicine.

- Will these side effects change your assessment whether to accept the treatment?
- Yes
- No

### Statistical analyses

Multiple logistic regression was used to analyse effects of gender, age, self-rated health status, experience with heart disease, education, income, price of medication and frequency of GP visits on willingness to accept treatment. Further, the effects of gender, age, self-rated health status, experience with heart disease and price of medication on continued willingness to accept treatment despite potential side effects were analysed among respondents initially accepting treatment. The analyses were adjusted for price, gender, age and experience with heart disease. The adjusted ORs are presented with 95% confidence intervals. Tests for trends were conducted when appropriate, i.e. for continuous and ordinal variables. Interactions between education and, respectively, experience with heart disease and self-rated health status were tested, as were interactions between price and, respectively, gender, age, experience with heart disease, self-rated health status, and income; and interactions between frequency of visits to the GP and, respectively, gender, age, experience with heart disease, self-rated health, and working-status (working/not working). Since the sensitivity to price differed depending on the income, the analyses on price were stratified on income levels. All analyses were conducted using Stata Release 11 (StataCorp, College Station, TX, USA).

## Results

### Sample characteristics

Of the 1069 respondents, 49% were females, and the average age of respondents was 51 years (Table 1). The self-rated health status was reported as good/very good by 62.5% of the respondents, fair by 30%, and poor/very poor by 7%. Slightly more than 5% had or had had a heart disease, while 24% had experience with family members with heart disease. Approximately 34% had a household income below 500 000 DKK (<80 000 USD), while 28% had a household income equal to or above 800 000 DKK (≥130 000 USD). The degree of educational attainment was classified into three categories with 23% having high school as their highest educational attainment, and 10% with at least a university degree (Table 1).

**Table 1:** **Sample characteristics**

	Participants
**Total, n**	**1069**
Gender, n (%)	
Female	521 (48.7)
Male	548 (51.3)
Age, mean (SD)	50.8 (5.8)
Age groups, n (%)	
40-44	180 (16.8)
45-49	278 (26.0)
50-54	267 (25.0)
55-60	344 (32.2)
Health status, n (%)	
Good/very good	668 (62.5)
Fair	325 (30.4)
Poor/very poor	75 (7.0)
Experience with heart disease, n (%)	
Yes, have or have had myself	55 (5.3)
Yes, family member has had	245 (23.6)
No	739 (71.1)
Household income, n (%)	
Low (<80,000 USD)	329 (34.2)
Medium	368 (38.2)
High (≥130,000 USD)	266 (27.6)
Education, n (%)	
Low (≤ high school)	243 (22.9)
Medium	717 (67.5)
High (university degree)	102 (9.6)

### Acceptance versus sample characteristics

Of the 1069 respondents, 365 (34%) accepted treatment before they were given information about potential side effects (Table 2). Gender did not affect willingness to accept treatment. Although the unadjusted analysis for trend indicated that acceptance of treatment increased with age (p = 0.03), the trend was no longer statistically significant when the analysis was adjusted for gender, prior experience with heart disease and price (p = 0.08) (Table 2). Personal experience with heart disease significantly affected the willingness to accept treatment (p < 0.01), whereas there was no difference in acceptance between respondents with no prior experience with heart disease and respondents with family members with heart disease (p = 0.17) (Table 2).

**Table 2:** **Willingness to accept treatment versus participant characteristics**

	Accepting treatment			
	Accepting, n (%)	OR _crude_	OR _adj_* (95%-CI)	Test for trend
**Total**	**365 (34.1)**	**-**	**-**	**-**
Gender		p = 0.309	p = 0.760	
Male	195 (35.6)	(ref.)	(ref.)	-
Female	170 (32.6)	0.87	0.96 (0.74, 1.25)	
Age groups		p = 0.158	p = 0.421	
40-44	52 (28.9)	(ref.)	(ref.)	p_crude_ = 0.027
45-49	91 (32.7)	1.20	1.10 (0.72,1.68)	p_adj_ =0.077
50-54	90 (33.7)	1.25	1.22 (0.80, 1.86)	
55-60	132 (38.4)	1.53	1.39 (0.93, 2.08)	
Health status		p = 0.282	p = 0.813	
Good/very good	216 (32.3)	(ref.)	(ref.)	p_crude_ = 0.118
Fair	119 (36.6)	1.21	1.07 (0.79, 1.43)	p_adj_ =0.529
Poor/very poor	29 (38.7)	1.31	1.16 (0.69, 1.97)	
Experience with heart disease		p < 0.001	p < 0.001	
Yes, have or have had myself	35 (63.6)	(ref.)	(ref.)	-
Yes, family member has had	87 (35.5)	0.31	0.33 (0.18, 0.62)	
No	232 (31.4)	0.26	0.27 (0.15, 0.48)	
Household income		p = 0.429	p = 0.522	
Low	114 (34.7)	(ref.)	(ref.)	p_crude_ = 0.497
Medium	120 (32.6)	0.91	0.96 (0.69, 1.33)	p_adj_ =0.428
High	100 (37.6)	1.14	1.16 (0.82, 1.65)	
Education		p = 0.037	p = 0.005	
Low	96 (39.5)	(ref.)	(ref.)	p_crude_ = 0.011
Medium	240 (33.5)	0.77	0.69 (0.50, 0.95)	p_adj_ = 0.001
High	26 (25.5)	0.52	0.42 (0.24, 0.73)	

*Adjusted for gender, age (four groups), experience with heart disease, price.

Neither self-rated health status nor household income affected the willingness to accept treatment (Table 2). Educational attainment significantly affected the willingness to accept treatment (p = 0.01). A significant trend of decreasing willingness to accept treatment with increasing educational attainment was observed (p < 0.01, following adjustment for price, gender, age, and experience with heart disease) (Table 2).

### Acceptance versus frequency of check-ups at the GP and price of treatment

The frequency of GP visits (2 times/month, 1 time/month, or 4 times/year) did not affect overall treatment acceptance (p = 0.90) (Table 3).

**Table 3:** **Willingness to accept treatment versus GP visits and price, stratified on household income**

	Accepting treatment			
	n (%)*	OR _crude_	OR _ad_(95%-CI)	Test for trend
Rate of GP visits		p = 0.905	p = 0.817**	
2 per month	125 (35.1)	(ref.)	(ref.)	p_crude_ = 0.676
1 per month	123 (33.9)	0.95	0.94 (0.68, 1.29)**	p_adj_ = 0.529
4 per year	116 (33.5)	0.94	0.90 (0.65, 1.25)**	
**Low income**				
Price per month	49 (46.7)	p < 0.001	p = 0.003***	
DKK 100		(ref.)	(ref.)	p_crude_ < 0.001
DKK 400	44 (35.8)	0.63	0.56 (0.32, 1.00)***	p_adj_ = 0.001
DKK 700	21 (21.0)	0.30	0.32 (0.17, 0.62)***	
**Medium income**				
Price per month		p = 0.052	p = 0.039***	
DKK 100	50 (40.7)	(ref.)	(ref.)	p_crude_ = 0.017
DKK 400	37 (30.8)	0.65	0.67 (0.39, 1.16)***	p_adj_ = 0.011
DKK 700	33 (26.4)	0.52	0.49 (0.28, 0.85)***	
**High income**				
Price per month		p = 0.020	p = 0.071***	
DKK 100	42 (44.7)	(ref.)	(ref.)	p_crude_ = 0.645
DKK 400	22 (22.6)	0.43	0.47 (0.25, 0.91)***	p_adj_ = 0.558
DKK 700	36 (41.9)	0.89	0.85 (0.46, 1.57)***	

*Number accepting treatment and percentage out of all subjects in the category.

**Adjusted for gender, age (four groups), experience with heart disease, in workforce, price.

***Adjusted for gender, age (four groups), experience with heart disease.

Tests for interaction showed that none of the following covariates interacted with frequency of check-ups at the GP: working status (p = 0.24), gender (p = 0.40), age (p = 0.16), health status (p = 0.17), experience with heart disease (p = 0.50) (Table 3).

Tests for interaction between price and various covariates with respect to the effect on willingness to accept treatment were conducted (Table 3). The results showed that household income interacted with price (p = 0.02), while none of the following covariates interacted with price: gender (p = 0.56), age (p = 0.99), self-rated health status (p = 0.53), experience with heart disease (p = 0.69). Further analyses on the effect of price on willingness to accept treatment were stratified on household income and demonstrated that price had a stronger impact on acceptance in the lower income groups (Table 3). For the two lowest income groups, there were significant trends of decreasing acceptance of the treatment when the price of the treatment was increased. This trend was not seen in the high income group (Table 3).

### Acceptance in view of presence of potential side effects following treatment

Among the 365 respondents accepting treatment in the first place, 156 (43%) stated that the information on potential side effect would not affect their original decision of accepting treatment (Table 4). None of the covariates considered had significant effect on continued acceptance of treatment in view of potential side effects (Table 4).

**Table 4:** **Continued acceptance of treatment in view of information on potential side effects**

	Continue to accept treatment despite side effects*			
	Accepting, n (%)	OR _crude_	OR _adj_** (95%-CI)	Test for trend
Total	156 (42.7)	-	-	-
Gender		p = 0.889	p = 0.832	
Male	84 (43.1)	(ref)	(ref)	-
Female	72 (42.4)	0.97	0.95 (0.62, 1.47)	
Age groups		p = 0.205	p = 0.257	
40-44	18 (34.6)	(ref)	(ref)	p_crude_ = 0.838
45-49	47 (51.7)	2.02	1.96 (0.96, 4.03)	p_adj_ = 0.960
50-54	37 (41.1)	1.32	1.23 (0.60, 2.54)	
55-60	54 (40.9)	1.31	1.38 (0.70, 2.74)	
Health status		p = 0.804	p = 0.682	
Good/very good	95 (44.0)	(ref)	(ref)	p_crude_ = 0.577
Fair	48 (40.3)	0.86	0.97 (0.43, 2.22)	p_adj_ = 0.458
Poor/very poor	12 (41.4)	0.90	1.24 (0.59, 2.58)	
Experience with heart disease		p = 0.656	p = 0.609	-
Yes, have or have had myself	14 (40.0)	(ref)	(ref)	
Yes, family member has had	34 (39.1)	0.96	0.81 (0.50, 1.31)	
No	103 (44.4)	1.20	0.86 (0.38, 1.97)	
Price		p = 0.402	p = 0.401	
DKK 100	69 (45.7)	(ref)	(ref)	p_crude_ = 0.725
DKK 400	43 (37.7)	0.72	0.71 (0.43, 1.18)	p_adj_ = 0.705
DKK 700	44 (44.4)	0.95	0.94 (0.56, 1.59)	

*Among the 365 (34.1%) subjects who originally accepted treatment.

**Adjusted for gender, age (four groups), experience with heart disease, price.

## Discussion

A study on prevalence and incidence of cardiovascular disease in Denmark has shown that the prevalence of cardiovascular disease among 45-66-year-old Danes is close to 8% in men as well as women [16], which fits reasonably well with our observation of 5.3% in the present study, considering that our population was slightly younger. Data from the general Danish population (drawn from Statistics Denmark) on average household income in the present age group have previously been reported to be approximately 100 000 USD, and close to 28% had high school as their highest educational attainment, while 7% had an education longer than 18 years, corresponding to a university degree [17]. This is in good agreement with the composition of our study group and supports its representativeness.

Persistence of statin use in primary prevention was recently reported to be 45% among patients in treatment [5]. Our respondents including both people with and without experience with heart disease had an overall acceptance rate of 34%. This low overall acceptance rate may to some extent be generated by the effect measure (an absolute risk reduction) applied in our study, as it has previously been demonstrated that information given in terms of absolute risk reduction is associated with lower appreciation of a clinical effect than relative risk reduction [17–19]. A more important reason when compared to the study by Chodick et al. [5] is that this study included patients in actual need of treatment, whereas the overall acceptance rate in our sample mainly included respondents with no personal experience with heart disease placed in a hypothetical situation. This is supported by the observation that respondents with personal experience with heart disease had an acceptance rate of 64% compared to 31% among those with no prior experience with heart disease.

Age has previously been reported to affect persistence in use of statins [6]. We observed this effect on acceptance of treatment in the crude results, but not after adjusting for gender and prior experience with heart disease. Previous findings may not have adjusted for prior experience with heart disease. The prevalence of heart disease increases with age, and age is included as an independent parameter in the Heart Score model used clinically to visualize risk levels and to guide patients and their GPs on initiating preventive treatment with statins or equivalent products. As seen in the present study, age may also in previous studies have acted as a proxy for previous experience with heart disease, without being independently associated with higher acceptance of treatment.

An interesting observation was that respondents with secondhand experience with heart disease (a family member had had heart disease) answered very much like respondents with no experience with heart disease. We had expected their response to more closely resemble respondents with personal experience with heart disease, but apparently respondents did not draw on family members’ experiences, when deciding to accept a personal treatment offer. A parallel observation has been seen in another recent study [13]. A possible explanation may be different perceptions of baseline risk across the respondent groups. For those with personal experience of heart disease, the presented baseline risk is most likely deemed irrelevant, as these patients perceive their risk to be significantly higher. In contrast, respondents with family members with known heart disease and potentially at increased risk may feel relieved by a stated risk as low as 1 in 10, and therefore feel less inclined to accept treatment in this theoretical scenario. This is not optimal as these respondents are known to have a higher risk of getting heart disease than others, and would potentially benefit from either treatment or lifestyle changes. A recent study in a setting with a spouse or parent being hospitalized suggest that this setting is a better opportunity to induce lifestyle changes among other family members [20].

Our *ex ante* hypothesis regarding the association between education and acceptance levels was that those with higher levels of education would exhibit lower time preference rates, and thus a greater willingness to engage in preventive health care. However, the results suggest that those with higher levels of education are much less willing to participate in the preventive programme. This observation may partly be explained by the observation from another study that time preference rates across different settings are highly uncorrelated (as suggested by van der Pol [12]). A different explanation might be that education is often associated with numeracy skills. Thus, those with higher levels of education may be better at understanding benefits given as percentages and therefore also at balancing risks against benefits. Perhaps only respondents with higher levels of education understood the benefit information sufficiently to realise that more than 90% of all patients will gain no extra benefit from the treatment.

In accordance with our pre-study hypothesis based on an expected decreasing marginal utility of income at higher income levels, the willingness to accept treatment was affected by the price of the treatment. Individuals with low income were more sensitive to the price of treatment than individuals with high income. This effect was present in the two lower income groups only. The price range was realistic and chosen sufficiently wide to demonstrate a possible effect of household income on the sensitivity to price. The present study based on hypothetical scenarios indicates that if the price of preventive medication increases above a certain threshold, a substantial proportion of the population may refrain from treatment. The actual threshold will depend on the income, and will therefore depend on the societal context and setting. The lowest monthly cost was equivalent to 80% of the minimal wage in Denmark for one hour unskilled work, which is about 120 DKK/hour.

We had hypothesised that the frequency of control visits to the GP would affect acceptance of treatment. This was not the case within the range of frequencies chosen by the authors. We are not able to conclude whether our respondents are generally insensitive to this cost of treatment, do not regard it as a treatment cost, or whether we have basically misjudged the frequency range that would cause a stratification of our population. Increasing the frequency of required visits to the GP might cause some respondents to refrain from treatment, but might also easily become clinically irrelevant. Moreover, for some people frequent visits to the GP may be interpreted as a benefit because closer surveillance may ensure detection of other risks, should they arise. An alternative explanation may be that the GP visits are interpreted as optional rather than mandatory.

Following the question on treatment acceptance based on information on risk reduction, price of medication, and frequency of GP visits, the respondents were faced with the presence of a risk of certain specified side effects. Following the information on side effects, 43% of the respondents who had accepted treatment answered that they would not change their initial decision. These respondents behave in line with a Cochrane review from 2008 stating that telling patients about adverse effects of treatment did not significantly affect their adherence [21], and a study on pain relief in osteoarthritis where patients demonstrated a willingness to accept some additional and specified risks to gain pain relief [22]. The same study, however, also showed that some side effects were regarded as more acceptable than others [22], and a recent study by Baroletti et al. [23] indicated that even fear of a potential side effect is an important factor for non-adherence. A possible reason for the discrepancy may be that the present study considers acceptance, which as previously discussed is one step earlier than adherence. Based on previous research on trade-offs between treatment effects and side effects [24], and a study on unspecific side effects related to lipid-lowering medication [25], we deliberately described the potential side effects as sufficiently grave to divide our population. The side effects may therefore appear slightly worse than what could be expected from a drug intended for lifelong treatment to prevent a disease not clinically present at the date of prescription. As slightly more than half of our respondents changed their initial decision to accept treatment in this hypothetical study, we succeeded in finding a level of adverse effect that allowed for analyses on potential determinants. We had expected that respondents with personal experience with heart disease or a poorer self-rated health status would accept treatment despite side effects to a higher degree than respondents with no prior experience with heart disease and with higher self-rated health. However, our results demonstrated that neither type of experience with heart disease nor low self-rated health status had a significant effect on continued acceptance of treatment in view of potential side effects. This is an important observation that deserves more detailed research, since some side effects appear very early after initiating medication, sometimes even before therapeutic effects, and diminish over time, whereas other side effects increase in frequency and severity with increasing treatment time. Further, most side effects very seldom occur, and a skewed assessment of treatment efficacy compared to risk of side effects may refrain some patients from treatment. Thus, more research is needed on medical risk communication to better allow patients to compare treatment efficacy with risk of side effects in quantitative terms.

### Strengths and weaknesses

A strength of the present study is that data were collected through the highly experienced organisation TNS Gallup taking advantage of their web-panel GallupForum, which is a validated data collection tool. Another strength is that the response rate was high (91%) among panel members who opened the web-based questionnaire. However, it is possible that some selection bias was introduced in connection with choosing to open the website at all. It is therefore reassuring that our respondent group is representative on sociodemographic characteristics. A further important strength is that we applied a sample design that was fully factorial with respect to the two treatment condition factors, price of medication and frequency of GP visits, which meant that the impact of the two types of costs could be studied independently. A weakness might be that no existing validated questionnaire suited the purpose of this study. However, care was taken in construction of the questionnaire. It was of priority to keep it short, and to ensure the questions were simple and unambiguous. In order to ensure that questions were understood by the respondents, we had the questionnaire pilot tested by Gallup.

## Conclusion

When presented with a hypothetical scenario, asked to imagine being at an increased risk of heart disease, and subsequently presented with an offer of a preventive medical intervention targeted to reduce the risk of heart disease, about one third of the study population was willing to accept the treatment offer. Respondents with personal experience with heart disease were more likely to accept treatment, while respondents with family members with heart disease were no more likely to accept than subjects with no prior experience with heart disease. The willingness to accept treatment was similar for both genders, and when adjusting for experience with heart disease, age was not associated with willingness to accept treatment. Socioeconomic status in terms of lower education was positively associated with acceptance. The price of the treatment reduced willingness to accept for the lower income groups, whereas it had no effect in the highest income group. The implication is that if the price of preventive medication increases above certain limits, a substantial proportion of the population may potentially refrain from treatment. Some 57% of respondents who were willing to accept treatment changed their decision following information on potential side effects. This is an important observation in relation to risk communication, since most side effects occur very seldom, and a skewed assessment of treatment efficacy compared to risk of side effects may refrain some patients from treatment. Thus, more research is needed to better allow patients to compare treatment efficacy with risk of side effects in quantitative terms.
